# Economic burden analysis of pediatric tracheobronchial foreign body

**DOI:** 10.3389/fpubh.2025.1546542

**Published:** 2025-03-28

**Authors:** Wen-yuan Wang, Tao Zhang, Wan-Yi Li, Shu-Ying Wang, Qi-Jun Zhao, Yong-Jun Wang

**Affiliations:** Pediatric Respiratory Department II, Gansu Provincial Maternity and Child-Care Hospital, Lanzhou, China

**Keywords:** economic burden, children, tracheobronchial foreign body, foreign body inhalation, urban–rural difference

## Abstract

**Objectives:**

Tracheobronchial foreign body aspiration (TFBA) constitutes a life-threatening pediatric emergency with substantial clinical and public health implications. While current research prioritizes diagnostic and therapeutic strategies for TFBA, limited attention has been paid to its socioeconomic consequences. This study focuses on Gansu Province, a representative underdeveloped region in China, to systematically assess both direct medical costs (surgical interventions and hospitalization) and broader socioeconomic impacts of pediatric TFBA management. The findings aim to inform evidence-based healthcare policies for childhood emergencies in resource-limited settings.

**Methods:**

Using Gansu Provincial statistical data, we analyzed the economic burden of 951 pediatric tracheobronchial foreign body cases (2017–2021) meeting inclusion criteria at a provincial tertiary hospital’s respiratory department.

**Results:**

(1) Urban–rural disparities in economic burden: The average annual total income of rural households was significantly lower than that of urban households (*p* < 0.01). The proportion of hospitalization costs relative to income in rural areas reached 36.31 ± 4.43%, 3.1 times that of urban households (11.91 ± 2.14%, *p* < 0.001). Rural minority-concentrated regions bore the heaviest burden (48.06%), while urban Han-majority regions had the lowest burden (9.29%). No significant urban–rural difference in surgical costs (*P*>0.05). (2) Regional heterogeneity in economic burden: Hospitalization costs in underdeveloped minority-concentrated rural areas reached 13,323¥ (8% higher than the rural average), yet their income (27,678¥) was 33% below the average. Their cost-to-income ratio (48.06%) was 3.2 times that of their urban counterparts (15.21%). In developed Han-majority regions, despite comparable hospitalization costs (11,872 ¥ vs. 12,339 ¥), the higher income (42% above average) resulted in the lowest cost-to-income ratio (21.52%).

**Conclusion:**

TFBA poses a critical global health challenge with disproportionate impacts on children aged 1–3 years and significant economic burden on families, especially in underdeveloped minority-concentrated rural areas. This study highlights severe urban–rural disparities in economic burdens.

## 1 Introduction

Tracheobronchial foreign body aspiration (TFBA) remains a significant pediatric emergency and global public health issue, particularly in children under 3 years old. It is associated with high morbidity, preventable mortality, and substantial economic burdens, primarily due to delayed diagnosis and complications such as airway obstruction or recurrent infections (1, 2). Previous research has shown that TFBA accounts for 7.9–18.1% of accidental injuries in children aged 0–14 years in China and 80% in children aged 1–3 years (3). TFBA are the third most common cause of death due to unintentional injury in children aged <1 year in the USA, where they have been reported to occur in 0.43 per 100,000 children aged <5 years (4). In recent years, the global incidence rate has not changed significantly. When compared to 1990, global incidence of TFBA in children under 5 years old decreased in 2019, but an increasing trend in the incidence rate of TFBA was observed from 2014 to 2019 (5). The diagnosis and differential diagnosis of TFBA remain clinically challenging due to non-specific or overlapping symptoms. A subset of cases is initially misdiagnosed as chronic pulmonary infections or wheezing disorders, causing patients to undergo multiple ineffective treatments and delayed interventions. In severe cases, complications such as airway necrosis or abscess formation may necessitate invasive procedures (e.g., thoracotomy), further exacerbating the risks of missed or incorrect diagnoses (6). These diagnostic pitfalls not only compromise clinical outcomes but also impose significant socioeconomic burdens on families and healthcare systems.

Current research on pediatric TFBA predominantly emphasizes diagnostic and therapeutic advancements, with scant attention paid to its socioeconomic burden, including healthcare expenditures and systemic costs. To address this gap, this study focuses on Gansu Province, a region with notable socioeconomic disparities, and retrospectively analyzes clinical data from children with TFBA treated at our institution over the past 5 \years. By quantifying direct medical costs (e.g., surgical and hospitalization expenses) and evaluating the financial strain on affected families, this investigation aims to delineate the broader economic impact of TFBA. These findings will serve as an evidence-based foundation for formulating targeted prevention strategies and optimizing public health resource allocation.

## 2 Methods

### 2.1 Research subjects

This study received ethical approval from the Institutional Review Board of our hospital. We retrospectively analyzed 951 pediatric patients with TFBA admitted to our center in Gansu Province between January 2017 and December 2021, adhering to established diagnostic criteria for foreign body aspiration. The cohort comprised 638 males (67.09%) and 313 females (32.91%), demonstrating male predominance (ratio 2.04:1). Geographic distribution revealed 664 rural cases (69.82%) versus 287 urban cases (30.18%), with a rural/urban ratio of 2.31:1. Exclusion criteria comprised endogenous airway foreign bodies, respiratory tract malformations, and comorbidities including cerebral palsy, congenital heart disease, or genetic metabolic disorders.

### 2.2 Economic burden assessment method

The economic burden of disease, a comprehensive metric introduced by the World Bank and the World Health Organization (WHO) in 1993, quantifies the health and socioeconomic impact of diseases through epidemiological and economic dimensions. The burden of disease (BOD) refers to the loss and impact on population health and social economy caused by a disease, disability, and premature death, including the epidemiological burden and economic burden of the disease (7, 8). The economic burden of disease comprises three components: direct costs (medical and non-medical expenditures during treatment), indirect costs (productivity losses due to illness), and intangible costs (non-monetizable psychosocial impacts). Direct costs encompass hospitalization fees, surgical expenses, and ancillary costs (e.g., transportation and accommodation for caregivers). Indirect costs primarily reflect productivity losses among caregivers, particularly in pediatric cases requiring prolonged family care. Intangible costs include psychological distress (e.g., caregiver anxiety) and reduced quality of life, which are challenging to quantify (9, 10).

In this study, we focus exclusively on direct medical costs (hospitalization and surgery expenses) for tracheobronchial foreign body (TFBA) management, excluding indirect expenditures (e.g., lost wages) and intangible burdens. Economic data were derived from the Gansu Statistical Yearbook (2017–2021) (11), including urban and rural household income statistics across the province to contextualize the financial impact relative to regional socioeconomic profiles.

### 2.3 Statistical methods

IBM SPSS Statistics 22 was used for data processing. Data were analyzed using IBM SPSS Statistics 22. Categorical variables were described as frequencies and percentages (%), with intergroup comparisons by χ^2^-test. Continuous variables were expressed as mean ± standard deviation (SD). Normality was assessed via the Kolmogorov–Smirnov test. For data conforming to a normal distribution, the t-test was used for comparison between two groups, and ANOVA was used for comparison among multiple groups. For data not conforming to a normal distribution, non-parametric tests were used. *p* < 0.05 was considered statistically significant, and *p*-value <0.01 as highly significant.

## 3 Results

### 3.1 Urban–rural disparities in economic burden

Gansu Province administers 14 prefecture-level divisions (12 cities and 2 autonomous prefectures) with 87 county-level administrative units. This study encompassed 951 pediatric TFBA cases from 72 counties across all municipal and autonomous prefectural jurisdictions, covering 82.76% of the province’s county-level administrative units.

Over the past 5 years (2017–2021), rural households in Gansu Province demonstrated a progressive increase in mean annual total income (33,603¥), while maintaining stable medical expenditure patterns: Surgical costs averaged 7,778¥, representing 23.02% of household income, with interannual variation peaking at 28.66% in 2018, dipping to 18.90% in 2021. Hospitalization costs averaged 12,244¥, representing 36.31% of household income, showing notable annual fluctuations ranging from 42.94% (2018) to 30.81% (2021). Detailed data are presented in Table 1.

**Table 1:** Urban–rural disparities in economic burden (¥).

Group	Year	2017	2018	2019	2020	2021	Mean ± SD
Rural	Family total income	28,405	29,881	34,339	36,397	38,997	33,603 ± 4,424
	Surgery cost	6,173	8,565	7,434	8,801	7,372	7,778 ± 1,866
	Proportion (%)	21.73	28.66	21.64	24.18	18.90	23.02 ± 3.28
	Hospitalization cost	10,275	12,832	11,819	13,542	12,018	12,244 ± 4,799
	Proportion (%)	36.17	42.94	34.42	37.21	30.81	36.31 ± 4.43
Urban	Family total income	78,431	79,649	98,858	106,667	111,611	95,043 ± 15,306
	Surgery cost	6,241	8,890	7,209	8,362	7,399	7,688 ± 1,853
	Proportion (%)	7.96	11.16	7.29	7.84	6.63	8.18 ± 1.75
	Hospitalization cost	9,756	12,249	10,332	12,003	11,205	11,176 ± 2,772
	Proportion (%)	12.43	15.38	10.45	11.25	10.04	11.91 ± 2.14

Values are presented with commas as thousand separators.

Urban households in Gansu Province experienced a steady rise in mean annual total income (95,043¥, 2017–2021), with surgical and hospitalization costs maintaining relative stability. Surgical costs averaged 7,688¥, accounting for 8.18% of household income, peaking at 11.16% in 2018 and declining to 6.63% in 2021. Hospitalization costs averaged 11,176¥ (11.91% of household income), ranging from 15.38% in 2018 to 10.04% in 2021. Detailed data are presented in Table 1.

Comparative analysis revealed statistically significant disparities between urban and rural households. The mean annual total income of urban families consistently exceeded that of rural counterparts across all study years (*p* < 0.01). While no significant intergroup difference was observed in surgical costs (*P*>0.05), rural areas demonstrated substantially higher hospitalization expenditures (*p* < 0.05). Notably, rural households bore disproportionately greater financial burdens, as evidenced by significantly elevated hospitalization-to-income ratios compared to urban populations (*p* < 0.05).

### 3.2 Regional heterogeneity in economic burden

To investigate the regional heterogeneity of economic burden associated with pediatric TFBA, this study implemented a multidimensional classification framework for Gansu Province’s 14 prefecture-level divisions. The categorization criteria integrated three key regional characteristics: economic development gradients (stratified by per capita GDP rankings), ethnic distribution patterns (measured through minority population concentration indices), industrial structural profiles (classified by dominant economic sectors). Four distinct regional typologies were subsequently identified, enabling systematic analysis of healthcare expenditure patterns. The economic burden of different regions in rural and urban areas was analyzed, with the results as shown in Table 2. Hospitalization costs and total family income are the average values from 2017 to 2021, all economic metrics were derived from the Gansu Statistical Yearbook (2017–2021 edition).

**Table 2:** Economic burden in different regions.

Development characteristics	Rural			Urban		
	Hospitalization cost	Total income	Proportion (%)	Hospitalization cost	Total income	Proportion (%)
Economically Developed Han-Majority Regions	11,872	58,595	21.52	11,305	121,760	9.29
Moderately Developed Multiethnic Regions	11,744	40,028	30.85	10,913	88,875	12.34
Less-Developed Ethnic Minority-Concentrated Regions	13,323	27,678	48.06	11,504	76,279	15.21
Resource-Dependent Transition Zones	12,287	43,540	31.20	11,689	105,096	11.16
Mean ± SD	12,339 ± 1,812	41,231 ± 15,309	33.84 ± 12.24	11,332 ± 1,047	95,799 ± 18,960	12.25 ± 2.69

Values are presented with commas as thousand separators.

Significant disparities in hospitalization cost-to-income ratios were observed across rural regions, ranging from 21.52% in Economically Developed Han-Majority Regions to 48.06% in Less-Developed Ethnic Minority-Concentrated Regions. Less-Developed Ethnic Minority-Concentrated Regions exhibited the most severe financial burden, with hospitalization costs averaging 13,323¥ (8% higher than the provincial rural mean), despite household incomes (27,678¥) 33% below the rural average. Economically Developed Han-Majority Regions demonstrated optimal economic resilience, combining the lowest cost ratio (21.52%) with incomes (58,595¥) 42% above rural averages, despite comparable hospitalization costs (11,872¥ vs. 12,339¥). Resource-Dependent Transition Zones showed intermediate burdens (31.20%), while Moderately Developed Multiethnic Regions reached 30.85%, exceeding urban counterparts by 2.4-fold (12.25 ± 2.69%). Full metrics are systematically compared in Table 2.

The urban economic burden analysis revealed hospitalization cost-to-income ratios ranging from 9.29% in Economically Developed Han-Majority Regions to 15.21% in Less-Developed Ethnic Minority-Concentrated Regions. Key urban–rural disparities included: Income Inequality: Urban households demonstrated 2.3-fold higher incomes than rural counterparts (95,799 ± 18,960¥ vs. 41,231 ± 15,309¥; *p* < 0.001). Cost Burden Gradient: Rural hospitalization costs consumed 33.84 ± 12.24% of household income – 2.8-fold higher than urban averages (12.25 ± 2.69%; p < 0.001). Less-Developed Ethnic Minority-Concentrated Regions: Despite lower absolute costs (11,504¥ vs. rural 13,323¥), Less-Developed Ethnic Minority-Concentrated Regions exhibited the highest urban burden (15.21%) due to depressed incomes (76,279¥ vs. urban mean 95,799¥). Detailed metrics are stratified by region in Table 2.

## 4 Discussion

### 4.1 Clinical and economic implications of TFBA

Unlike ordinary respiratory infections, pediatric TFBA cases require mandatory surgical intervention, incurring substantially higher hospitalization expenses. Diagnostic delays and misdiagnoses, particularly when resulting in extended foreign body retention due to missed identification or delayed clinical intervention, exacerbate clinical complexity, leading to escalated therapeutic costs and disproportionate family financial strain (12). This study systematically evaluates the economic burden of clinically confirmed TFBA cases. While healthcare-related economic burdens manifest multidimensionally, our analysis focuses on quantifiable direct medical costs: surgical expenditures and hospitalization fees. Given that surgical costs constitute the predominant financial component in foreign body management, these are subjected to dedicated stratified analysis.

### 4.2 Urban–rural disparities in economic burden

Our study reveals significant disparities in the economic burden of pediatric TFBA between urban and rural areas in Gansu Province, reflecting broader socioeconomic inequities. Rural households face disproportionate financial strain, with an average annual total income (33,603¥) representing only 35.3% of urban households’ income (95,043¥), while rural hospitalization costs consume 36.31% of total family income—3.1 times the urban rate (11.91%, *p* < 0.001). Surgical costs account for nearly a quarter (23.02%) of rural household income, compared to <10% in urban areas. This disparity stems from three compounding factors, income disparity: A 2.8-fold urban–rural income gap (urban 95,043¥ vs. rural 33,603¥); Healthcare cost differentials: rural hospitalization costs exceed urban levels by 9.6% (12,244¥ vs. 11,176¥). Cumulative effects of diagnostic delays: rural cases exhibit wider annual fluctuations in hospitalization cost-to-income ratios (30.81–42.94% vs. urban 10.04–15.38%), likely due to delayed referrals and higher complication rates prolonging hospital stays (13, 14). The inverse relationship between regional economic development and healthcare affordability highlights systemic vulnerabilities in rural healthcare systems. Lower incomes exacerbate these disparities: despite earning 2.8 times less, rural households shoulder significantly higher hospitalization and surgical expenditures (*p* < 0.05). Diagnostic delays and limited access to timely interventions likely amplify clinical complexity, driving cost escalation in underserved areas (15).

### 4.3 Regional heterogeneity in economic burden

Regional analysis based on a multidimensional classification framework (economic development, ethnic distribution, and industrial structure) reveals that underdeveloped ethnic minority-concentrated regions endure a dual burden of “low income-high expenditure”: Rural hospitalization costs (¥13,323) exceed the provincial rural average by 8%, while household income (¥27,678) falls 33% below the mean, yielding a cost-to-income ratio of 48.06%—3.2 times higher than urban counterparts. In contrast, economically developed Han-majority regions demonstrate economic resilience: despite comparable hospitalization costs (¥11,872 vs. provincial average ¥12,339), their household income (¥58,595, 42% above rural averages) reduces the cost-to-income ratio to 21.52%.

This “burden inversion” phenomenon stems from cultural-cognitive disparities, healthcare resource mismatches, environmental exposures. Cultural-cognitive disparities: the interplay between economic development and cultural-cognitive factors critically shapes health outcomes. In economically underdeveloped ethnic minority regions, limited health literacy and entrenched traditional medical practices hinder timely recognition and management of foreign body aspiration (FBA) (16). Healthcare resource mismatches: Medical staff in primary hospitals have limited ability to identify foreign bodies, high misdiagnosis rate at first diagnosis, and low bronchoscope allocation rate, which leads to multiple levels of referral to specialized hospitals and delays in treatment time. Environmental exposures: Geographical disparities further amplify risks, rural agricultural communities exhibit higher exposure to plant-based foreign bodies (e.g., sunflower seeds, peanuts), particularly during harvest seasons when children lack adequate supervision, parental risk awareness remains suboptimal (17). Globally, children in low-and middle-income countries face amplified TFBA risks due to overlapping vulnerabilities: socioeconomic deprivation, limited healthcare access, and insufficient risk awareness. These systemic inequities transform a preventable condition into a cascade of medical and financial crises, demanding culturally tailored prevention strategies and resource redistribution (18).

### 4.4 Control strategy

TFBA imposes catastrophic health expenditures across both urban and rural households, with hospitalization costs consuming a substantial proportion of family income—particularly in socioeconomically disadvantaged minority-concentrated regions. To address this inequity, a multi-tiered intervention strategy is imperative: (1) Targeted Medical Subsidy Mechanisms, the concentration of high hospital costs in low-income areas highlights a vicious cycle in which poverty increases health spending and further exacerbates economic disadvantage, and targeted health subsidies are recommended. (2) Strengthening Primary Care Diagnostic Capacity, portable bronchoscopes were provided in county-level hospitals to strengthen the ability of grassroots children’s respiratory doctors to identify, diagnose and deal with children’s TFBA in emergencies. (3) Building a health education system: Foreign body prevention is more important than treatment, and primary health care training and public awareness campaigns should be strengthened (18).

### 4.5 Study limitations

This study has several limitations that warrant consideration. First, although our institution pioneered bronchoscopic interventions in the province and manages the majority of TFBA cases, recent advancements in endoscopic techniques have enabled municipal hospitals to initiate similar services. Consequently, our single-center data may not fully represent provincial epidemiological patterns, further multi-center, large-sample studies are needed. Second, the economic analysis focused solely on direct medical costs, excluding indirect expenditures such as caregiver lost wages, transportation, and accommodation expenses. Therefore, the overall economic burden caused by TFBA is more than this. Third, our use of regional average household incomes rather than individual family-level data may obscure socioeconomic heterogeneity, making the assessment of family economic burden not objective enough. In addition, it is difficult to obtain the intangible costs caused by the spiritual burden of grief, anxiety, and inconvenience of children and their caregivers in actual research, and these indirect economic losses may have a significant impact on the total costs, which need to be further investigated in future research.

## 5 Conclusion

TFBA remains a critical yet preventable global pediatric health challenge, disproportionately affecting children aged 1–3 years. As a time-sensitive iatrogenic crisis, TFBA causes acute respiratory compromise and chronic complications including obstructive pneumonia atelectasis, mortality in delayed diagnoses, and a heavier financial burden. This study systematically reveals the urban–rural disparities and regional heterogeneity in the economic burden of TFBA among children in Gansu Province, highlighting the structural incongruities between healthcare resource allocation and imbalanced socioeconomic development in China’s underdeveloped regions. Our findings reinforce three essential pillars for TFBA management, first, early intervention imperative—intervention within 24 h significantly reduces both complication risks and treatment costs. Second, socioeconomic equity focus—rural minority communities exhibit catastrophic health expenditure rates than urban counterparts, necessitating targeted medical subsidy programs. Third and foremost, prevention as cost-effective strategy, majority of cases can be prevented through measures such as caregiver education and toy safety regulations. In essence, preemptive public health measures outweigh reactive clinical management in averting this avoidable childhood trauma.

## Data Availability

The original contributions presented in the study are included in the article/supplementary material, further inquiries can be directed to the corresponding author.
